# Urinary endogenous sex hormone levels and the risk of postmenopausal breast cancer

**DOI:** 10.1038/sj.bjc.6600890

**Published:** 2003-04-29

**Authors:** N C Onland-Moret, R Kaaks, P A H van Noord, S Rinaldi, T Key, D E Grobbee, P H M Peeters

**Affiliations:** 1 Julius Center for Health Sciences and Primary Care, University Medical Center Utrecht, Room D-01.335, PO Box 85500, 3508 GA Utrecht, The Netherlands; 2 Hormones and Cancer Group, International Agency for Research on Cancer (IARC), 150 cours Albert Thomas, 69372 Lyon, France; 3 Cancer Research UK Epidemiology Unit, University of Oxford, Gibson Building, Radcliffe Infirmary, Oxford OX2 6HE, UK

**Keywords:** urinary sex steroids, endogenous hormones, breast cancer risk, prospective study

## Abstract

To assess the relation between urinary endogenous sex steroid levels and the risk of postmenopausal breast cancer, a nested case–cohort study was conducted within a large cohort (the DOM cohort) in the Netherlands (*n*=9 349). Until the end of follow-up (1 January 1996), 397 postmenopausal breast cancer cases were identified and a subcohort of 424 women was then taken from all eligible women. Women using hormones were excluded, leaving 364 breast cancer cases and 382 women in the subcohort for the analyses. Concentrations of oestrone, oestradiol, testosterone, 5*α*-androstane-3*α*, 17*β*-diol and creatinine were measured in first morning urine samples, which had been stored since enrolment at −20°C. A Cox proportional Hazards model was used, with Barlow's adjustment for case–cohort sampling, to estimate breast cancer risk in quartiles of each of the, creatinine corrected, hormone levels, the lowest quartile being the reference group. Women with higher levels of all four of the hormones were at increased risk for postmenopausal breast cancer (highest *vs* lowest quartile: incidence rate ratio for oestrone (IRR_oestrone_=2.5, 95% CI: 1.6–3.8; IRR_oestradiol_=1.5, 95% CI: 1.0–2.3; IRR_testosterone_=1.6, 95% CI: 1.0–2.4; IRR_5*α*-androstane-3*α*, 17*β*-diol_=1.7, 95% CI: 1.1–2.7). In conclusion, women with higher excretion levels of both oestrogens and androgens have an increased risk of breast cancer.

Established risk factors for breast cancer, such as early age at menarche, late age at menopause, late age at first full-term pregnancy or nulliparity, indicate that endogenous sex hormones play an important role in breast cancer aetiology (Bernstein and Ross, 1993).

For almost 30 years now, several case–control and cohort studies addressed this topic. Recently, a pooled analysis of nine prospective studies, including 663 breast cancer cases and 1765 controls, showed that postmenopausal women in the highest quintiles of oestrone-, oestradiol- and testosterone concentrations in blood had a statistically significant two-fold increased risk of breast cancer (The Endogenous Hormones and Breast Cancer Collaborative Group, 2002). However, this pooled analysis was based on nine relatively small prospective studies, which in themselves did not show consistent results, probably because of lack of power (Garland *et al*, 1992; Helzlsouer *et al*, 1994; Toniolo *et al*, 1995; Berrino *et al*, 1996; Dorgan *et al*, 1996; Thomas *et al*, 1997; Hankinson *et al*, 1998; Cauley *et al*, 1999; Kabuto *et al*, 2000). And in the five largest of these studies, the median time to diagnosis was less than 3 years, while the other four had much longer follow-up (The Endogenous Hormones and Breast Cancer Collaborative Group, 2002).

To further evaluate the relation between urinary endogenous sex steroids (i.e. oestrone, oestradiol, testosterone and 5*α*-androstane-3*α*, 17*β*-diol) and the risk of postmenopausal breast cancer, we conducted a nested case–cohort study in a large population-based prospective cohort study in the Netherlands.

## METHODS

### Subjects

All women born between 1911 and 1945 living in Utrecht and surroundings were invited to participate in a large population-based screening programme for early detection of breast cancer (the DOM-cohort) between 1975 and 1986 (de Waard *et al*, 1984). All participants were asked to fill in a lifestyle questionnaire containing questions regarding breast cancer-risk factors, medical history, exogenous hormone use and menopausal status. Also, anthropometric measurements (e.g. height, weight) were taken and women were asked to donate a first morning urine sample on the day of their examination. Urine samples were then stored at −20°C in 250 ml plastic polypropylene jars, without preserving agents, until analysis. A total of 27 718 women participated in this cohort.

Women who were naturally postmenopausal at recruitment (defined as no menstrual period for at least 12 months, after spontaneous cessation of their menses) and who had no history of breast cancer were eligible for the present study (*n*=9349). All women were followed until 1st January 1996 for the occurrence of breast cancer through their general practitioners and, from 1986 onwards, through linkage with the regional cancer registry. A nested case–cohort study was designed to study the relation between endogenous sex steroids and the risk of postmenopausal breast cancer. Until the end of follow-up, 397 breast cancer cases were identified and a random sample of 424 women (the subcohort) was taken from all eligible women. A urine sample could not be found for three cases and three women from the subcohort. Women using hormone replacement therapy or oral contraceptives at the time of urine sampling were excluded (30 cases and 38 women from the subcohort). Urine samples were sent to the International Agency for Research on Cancer (IARC), Lyon, France, for hormonal measurements. One sample was lost during the analyses and was excluded, leaving 364 cases and a subcohort of 382 women for the analyses.

### Hormone measurement

Urine samples were analysed at the IARC in Lyon, France. Laboratory technicians were blinded as to the disease status of the samples. Equal numbers of samples taken from the case group and from the subcohort were analysed together within batches of 22 urine samples. The hormone metabolites, oestrone (E1), oestradiol (E2), testosterone (TST) and 5*α*-androstane-3*α*, and 17*β*-diol (3*α*D) were measured by radioimmunoassay (RIA) after enzymatic hydrolysis, solid phase extraction and high-performance liquid chromatography (HPLC) purification of the urine samples. Results were expressed in nanogram analyte per litre. The method used in this study has been described in detail elsewhere (Rinaldi *et al*, 2003). Intra- and interassay coefficients of variation were 8.7 and 17.2% for E1, 12.2 and 14.8% for E2, 8.3 and 15.3% for TST and 9.0 and 11.4% for 3*α*D. Reproducibility of these assays in urine was tested in a random sample of 45 postmenopausal women from the DOM cohort, who delivered urine samples at three different examinations of breast cancer screening and who did not use exogenous hormones. The average time interval between the first and second urine collection was 1.1 years (0.9–4.4 years) and between the second and the third collection was 4.0 years (1.3–7.7 years). Intraclass correlation coefficients for sample replicates were above 0.93 for all four hormone metabolites, indicating good reproducibility from a laboratory error perspective. The intraclass correlation coefficients over time were 0.59 for E1, 0.62 for E2, 0.85 for TST and 0.55 for 3*α*D, indicating reasonable stability of women's urinary hormone levels over several years (Rinaldi *et al*, 2003).

Creatinine was measured in each sample by kinetic Jaffé reaction (Hitachi 717, Roche, Central laboratory for Biochemistry, Hôpital de l'Antiquaille, Lyon, France).

### Data analyses

To correct for between-subject variability in urine dilution, in each sample the hormone concentration (expressed in ng/l) was divided by the creatinine concentration (expressed in mg/l). All analyses included creatinine-adjusted hormone levels.

Concentrations of all four hormone-metabolites were logarithmically transformed to produce approximately normal distributions. Geometric mean values and their 95% confidence intervals were then calculated in cases and in the subcohort.

Women were classified into quartiles of concentrations of E1, E2, TST and 3*α*D, based on the distribution of the creatinine-corrected hormone levels in the subcohort. Women with hormone levels below the detection limit (E1: *n*=5; E2: *n*=4; TST: *n*=1; 3*α*D: *n*=0) were included in the lowest quartile. Incidence rate ratios (IRR) were calculated in each quartile compared to the first, using a Cox Proportional Hazards Model with Barlow's weighting method (Barlow *et al*, 1999). Family history (yes/no), defined as at least a mother or one sister diagnosed with breast cancer, smoking (ever, never), parity (nulliparous/parous), time since menopause, age at menopause, age at first full-term pregnancy (no children, <25, 25–29, >29) and socioeconomic status (SES), based on type of health insurance (low: public health insurance; intermediate: civil servants plan; high: private insurance) were evaluated for confounding. If the IRR changed by 10% or more when one of these variables was included, the variable was then included in the final model to adjust the IRR. Body mass index (BMI) is often considered to be in the aetiological pathway, because in postmenopausal women oestrogens are mainly formed through the aromatisation of androgens in fatty tissue. Therefore, IRRs with and without adjustment for BMI are presented. Elevated breast cancer risks found for androgens may be caused by aromatisation of androgens into oestrogens. To check whether androgens have an independent effect on breast cancer risk, we calculated standardised values for each of the four hormone metabolites. We then added the standardised values of the two oestrogens and classified women according to high or low oestrogen levels along the median (cutoff: −0.2672). The same procedure was followed for the androgens (cutoff: −0.3883). Subsequently, a new variable was calculated, clustering women into four categories, according to their combined level of exposure to oestrogens and androgens, that is, both low, low in oestrogens and high in androgens and *vice versa*, or high in both oestrogens and androgens. IRRs were calculated for each category compared to women who were low for both (standardised) oestrogen- and androgen levels.

To evaluate the effect of latent disease at urine collection on breast cancer risk, we studied the association between levels of hormone concentrations and the risk for breast cancer for those women who were free of breast cancer for at least 2 years after urine sample collection. To evaluate the influence of the time interval between urine collection and the diagnosis, analyses were done separately for women who developed breast cancer within the first 9 years of follow-up and after 10 years. Linear trends were tested by including quartile scores 1, 2, 3 and 4 as a continuous variable in the model.

Cox Proportional Hazards models were performed using the SAS macro described by Barlow *et al* (1999). For all other statistical analyses, the Statistical Package for Social Sciences (SPSS) was used.

## RESULTS

The median follow-up in the subcohort was 19 years (range 0–21 years). Compared to the subcohort, women with breast cancer more often reported a positive family history of breast cancer and were slightly heavier (Table 1

**Table 1:** Characteristics of the study population

	**Subcohort (*n*=382)**	**Breast cancer cases (*n*=364)**
Age at intake (median, range)	58 (43–66)	58 (41–65)
Age at menopause (median, range)	50 (37–60)	50 (36–60)
Years since menopause (median, range)	7 (0–25)	7 (0–26)
Age at menarche (median, range)	13.3 (10.2–18.0)	13.0 (10.5–18.0)
Nulliparous	81 (21.2%)	65 (17.9%)
If parous: number of children (median, ranged)	3 (1–9)	2 (1–9)
Age at first full-term pregnancy (median, range)	27 (18–42)	27 (16–41)
Positive family history of breast cancer^a^	28 (7.4%)	54 (15.2%)
Ever smoking	99 (25.9%)	91 (25.6%)
Body mass index (kg m^−2^) (mean, s.d.)	26.1 (4.2)	26.8 (4.1)
Height (cm) (mean, s.d.)	162.1 (6.1)	162.5 (6.2)
Weight (kg) (mean, s.d.)	68.4 (11.1)	70.8 (11.6)
*SES*		
* *Low	263 (68.8%)	525 (69.2%)
* *Medium	41 (10.7%)	43 (11.8%)
* *High	78 (20.4%)	69 (19.0%)
^a^At least one first-degree relative with breast cancer.			Percentage of missing values varied from 0 to 1.3% per variable, except for age at menarche, which was only asked in a subgroup (147 breast cancer cases and 198 in subcohort).		

). Case women showed higher geometric mean levels of all four hormone-metabolites at enrolment, although none of these differences were statistically significant (Table 2

**Table 2:** Geometric means and 95% confidence intervals for levels of oestrone, oestradiol, testosterone and 5*α*-androstane-3*α*, 17*β*-diol in breast cancer cases and in the subcohort

	**Subcohort**		**Breast cancer cases**	
	**Geometric mean**	**95% CI**	**Geometric mean**	**95% CI**
Oestrone (ng mg^−1^ creatinine)	1.76	0.46–6.68	1.98	0.56–6.95
Oestradiol (ng mg^−1^ creatinine)	0.53	0.13–2.12	0.58	0.16–2.15
Testosterone (ng mg^−1^ creatinine)	2.64	0.64–10.84	2.94	0.75–11.45
5*α*-androstane-3*α*, 17*β*-diol (ng mg^−1^ creatinine)	21.87	6.31–75.74	24.16	7.79–74.91

).

Table 3

**Table 3:** Breast cancer risks for quartiles of oestrone, oestradiol, testosterone and 5*α*-androstane-3*α*, 17*β*-diol

	**Women in subcohort (*n*=382)**	**Breast cancer cases (*n*=364)**	**IRR^a^**	**95% CI**	**IRR^b^**	**95% CI**
*Oestrone*^a^						
* *0.06–1.19 ng mg^−1^	95	57	1.0	—	1.0	—
* *1.19–1.77 ng mg^−1^	95	95	1.9	1.2–3.0	1.9	1.2–3.0
* *1.78–2.50 ng mg^−1^	95	79	1.2	0.8–2.0	1.2	0.8–2.0
* *>2.50 ng mg^−1^	95	132	2.5	1.6–3.8	2.4	1.5–3.8
*P*_trend_				0.001		0.002
						
*Oestradiol*						
* *0.08–0.34 ng mg^−1^	95	77	1.0	—	1.0	—
* *0.34–0.49 ng mg^−1^	96	79	1.1	0.7–1.7	1.1	0.7–1.7
* *0.49–0.77 ng mg^−1^	96	101	1.4	0.9–2.1	1.4	0.9–2.1
* *>0.77 ng mg^−1^	95	107	1.5	1.0–2.3	1.5	1.0–2.3
*P*_trend_				0.03		0.03
						
*Testosterone*						
* *0.03–1.85 ng mg^−1^	96	80	1.0	—	1.0	—
* *1.86–2.71 ng mg^−1^	95	78	1.1	0.7–1.7	1.1	0.7–1.7
* *2.72–4.16 ng mg^−1^	96	97	1.5	0.9–2.3	1.5	1.0–2.3
* *>4.16 ng mg^−1^	95	109	1.6	1.0–2.4	1.6	1.0–2.4
*P*_trend_				0.02		0.02
						
*5α-androstane-3α, 17β-diol*^*c*^						
* *0.85–15.93 ng mg^−1^	95	62	1.0	—	1.0	—
* *15.94–23.67 ng mg^−1^	96	103	1.7	1.1–2.7	1.7	1.1–2.7
* *23.68–33.65 ng mg^−1^	96	101	1.7	1.1–2.6	1.7	1.1–2.6
* *>33.65 ng mg^−1^	95	97	1.7	1.1–2.7	1.8	1.1–2.8
*P*_trend_				0.03		0.03
^a^Adjusted for family history of breast cancer (yes/no). ^b^Additional adjustment for BMI. ^c^Measurements for oestrone were missing in one case and two women from the subcohort. Measurements for 5*α*-androstane-3*α*, 17*β*-diol was missing for one case. IRR, incidence rate ratio.						

shows the IRRs for each quartile of levels of E1, E2, TST and 3*α*D adjusted for family history of breast cancer. Compared to the lowest quartiles of oestrogens (E1 and E2), women with higher levels of these metabolites were found to be at higher risk of developing breast cancer (highest *vs* lowest quartile: IRR_E1_=2.5, 95% CI: 1.6–3.8; IRR_E2_=1.5, 95% CI: 1.0–2.3). Tests for trend were statistically significant for both oestrogens.

Also, women with higher levels of both testosterone and 3*α*D had an increased risk of breast cancer (highest *vs* lowest quartile: IRR_TST_=1.6, 95% CI: 1.0–2.4; IRR_3*α*D_=1.7, 95% CI: 1.1–2.7), with statistically significant tests for trend. Analysis of hormone levels unadjusted for creatinine, showed similar results, but relative risks were slightly smaller and not all statistically significant anymore.

Additional adjustment for BMI did not change the risks associated with any of the hormone metabolites (Table 3). Excluding from the analysis women with a diagnosis within the first 2 years after urine collection did not alter the results (data not shown).

Table 4

**Table 4:** Correlation coefficients for the correlation between the concentrations of each of the four hormones

	**Oestrone**	**Oestradiol**	**Testosterone**	**5*α*-androstane-3*α*, 17*β*-diol**
Oestrone	1.0	0.93^**^	0.10^*^	0.08^*^
Oestradiol 2		1.0	0.13^**^	0.11^**^
Testosterone			1.0	0.53^**^
5*α*-androstane-3*α*, 17*β*-diol				1.0
^*^P<0.05; ^**^P<0.01.				

shows that all four hormone metabolites were correlated with each other. We tried to disentangle the effects of oestrogens and androgens by classifying women into four categories. Table 5

**Table 5:** Risk of breast cancer for high levels of oestrogens and androgens

**Levels of oestrogen/androgen**^a^	**Women in subcohort (*n*=380)**	**Breast cancer cases (*n*=362)**	**IRR**	**95% CI**
Low/low	143	113	1.0	—
Low/high	63	53	1.4	0.9–2.3
High/low	60	58	1.4	0.9–2.3
High/high	114	138	1.7	1.1–2.4
^a^Based on the median of the standardised values of each of the hormone levels. IRR, incidence rate ratio.				

shows that, independent from the level of total oestrogen, women with high levels of androgens are at increased risk for breast cancer (IRR=1.4; 95% CI: 0.9–2.3). If women have high levels of both oestrogens and androgens, the risk of breast cancer is slightly higher compared to the risk of oestrogens and androgens separately (IRR=1.7; 95% CI: 1.1–2.4), although this difference was not statistically significant.

Table 6

**Table 6:** Risks of breast cancer for quartiles of hormone levels stratified for the time period between urine collection and diagnosis of breast cancer (<10 years; ⩾10 years)

		**Time interval between urine collection and diagnosis of breast cancer**					
		**<10 years**			**≥10 years**		
	**Women in subcohort (*n*=382)**	**Breast cancer cases (*n*=184)**	**IRR^a^**	**95% CI**	**Breast cancer cases (*n*=180)**	**IRR^a^**	**95% CI**
*Oestrone*							
0.06–1.19 ng mg^−1^	95	31	1.0	—	26	1.0	—
1.19–1.77 ng mg^−1^	95	40	1.5	0.8–2.7	55	2.2	1.3–4.0
1.78–2.50 ng mg^−1^	95	43	1.2	0.7–2.2	36	1.2	0.7–2.2
>2.50 ng mg^−1^	95	69	2.6	1.5–4.6	63	2.3	1.3–4.0
*P*_trend_				0.002			0.04
							
*Oestradiol*							
0.08–0.34 ng mg^−1^	95	38	1.0	—	39	1.0	—
0.34–0.49 ng mg^−1^	96	34	0.9	0.5–1.6	45	1.3	0.7–2.2
0.49–0.77 ng mg^−1^	96	55	1.4	0.8–2.4	46	1.3	0.8–2.3
>0.77 ng mg^−1^	95	57	1.9	1.1–3.3	50	1.2	0.7–2.1
*P*_trend_				0.007			0.45
							
*Testosterone*							
0.03–1.85 ng mg^−1^	95	39	1.0	—	41	1.0	—
1.86–2.71 ng mg^−1^	96	51	1.2	0.7–2.2	37	1.0	0.6–1.7
2.72–4.16 ng mg^−1^	96	55	1.4	0.8–2.5	57	1.5	0.9–2.6
>4.16 ng mg^−1^	95	40	2.1	1.2–3.5	45	1.2	0.7–2.1
*P*_trend_				0.009			0.24
							
*5α-androstane-3α, 17β-diol*							
0.85–15.93 ng mg^−1^	97	29	1.0	—	33	1.0	—
15.94–23.67 ng mg^−1^	94	52	1.8	1.0–3.2	51	1.6	0.9–2.8
23.68–33.65 ng mg^−1^	96	46	1.6	0.9–2.9	55	1.8	1.0–3.0
>33.65 ng mg^−1^	95	57	2.3	1.3–4.1	40	1.3	0.8–2.4
*P*_trend_				0.01			0.26
^a^Adjusted for family history of breast cancer (yes/no). IRR, incidence rate ratio.							

shows the risk of breast cancer in quartiles of each of the four hormones stratified according to the time interval between urine collection and the diagnosis of breast cancer cases. Especially in the highest quartile of each of the four hormones, the risks seem to be somewhat diluted if the periods between urine collection and the diagnosis of breast cancer was over 9 years. Except for oestrone, tests for trend were no longer statistically significant. If the period between urine collection and the diagnosis of breast cancer was less than 10 years, women with elevated hormone levels showed increased risk. Tests for trend were statistically significant for all four hormone metabolites (Table 6).

## DISCUSSION

The present prospective study shows that postmenopausal women with higher excretion levels of oestrogens as well as androgens have an increased risk of breast cancer.

Certain aspects of the study need to be considered. The number of women who developed breast cancer was fairly large (*n*=364) and follow-up was long (median time to diagnosis=9.8 years). The largest prospective study so far published included 156 cases and a median time to diagnosis of 2.6 years (Hankinson *et al*, 1998), while the study with the longest follow-up (median=12 years) included 29 breast cancer cases and 58 controls (Helzlsouer *et al*, 1994; The Endogenous Hormones and Breast Cancer Collaborative Group, 2002). A possible disadvantage of a short follow-up is that breast tumours occurring during follow-up may have already been present at the time of enrolment in the study. Recent evidence shows that, apart from possible promotion of breast cancer through the stimulation of cell proliferation, circulating oestrogens might also act in the initiation. This initiation might occur either because oestrogens increase the mitotic activity and therefore the probability that DNA damage is not repaired and converted into DNA mutations (Pike *et al*, 1993), or through the formation of oxidative metabolites, which can directly form DNA adducts (Yager, 2000). Possible effects on initiation of the tumour cannot be picked up in studies with short follow-up. Our study shows that the risk of breast cancer was clearly increased in women who developed breast cancer in the first 9 years of follow-up. These results suggest that endogenous sex steroids have their effect predominantly on the promotion of breast tumours, which is in line with several other observations, such as the rather rapid effect of menopause on breast cancer risk (Pike *et al*, 1993). However, we cannot completely exclude the possibility that there may be an early stage effect as well, since increased risks were also observed for women diagnosed more than 9 years after urine collection. This would be in line with the long-lasting effects of, for instance, age at menarche (Baanders and de Waard, 1992; Peeters *et al*, 1995).

In blood, oestrogens as well as androgens are bound to sex hormone binding globulin and albumin, and it has been suggested that only unbound sex steroids could have their effects in breast epithelial cells (Bernstein and Ross, 1993; Pike *et al*, 1993). We classified women according to their hormone levels in urine. Measurements in urine reflect metabolised amounts of hormones, but it is not clear how these levels relate to biologically active levels in breast tissue. Therefore, using urine levels to classify women's exposure to endogenous sex steroids could lead to misclassification. However, because disease had not yet occurred at the time of urine collection, misclassification is likely to have been nondifferential and therefore to have led to an underestimation of the risk.

The risks for breast cancer associated with oestrogen levels found in the present study are in concordance with results from most other studies. Until now, 10 other prospective studies have been published on oestrogen levels in either serum or urine (Garland *et al*, 1992; Helzlsouer *et al*, 1994; Toniolo *et al*, 1995; Berrino *et al*, 1996; Dorgan *et al*, 1996; Key *et al*, 1996; Thomas *et al*, 1997; Hankinson *et al*, 1998; Cauley *et al*, 1999; Kabuto *et al*, 2000). Eight of these studies showed a positive association of oestradiol with breast cancer risk in postmenopausal women (Toniolo *et al*, 1995; Berrino *et al*, 1996; Dorgan *et al*, 1996; Key *et al*, 1996; Thomas *et al*, 1997; Hankinson *et al*, 1998; Cauley *et al*, 1999; Kabuto *et al*, 2000), although statistically significant in only four (Toniolo *et al*, 1995; Thomas *et al*, 1997; Hankinson *et al*, 1998; Cauley *et al*, 1999). Most of the prospective studies included a small number of cases (24–156 cases per study).

Also, for testosterone, we observed an increased risk for breast cancer. Prospective studies showed an increased risk for breast cancer for the aromatisable androgens (testosterone and androstenedione) in most cases (Berrino *et al*, 1996; Dorgan *et al*, 1996; Thomas *et al*, 1997; Zeleniuch-Jacquotte *et al*, 1997; Cauley *et al*, 1999), but not statistically significant in all (Helzlsouer *et al*, 1994; Hankinson *et al*, 1998). In one study, no increased risk was observed (Garland *et al*, 1992).

The estimates for breast cancer risk in the highest quartiles of oestrone, oestradiol and testosterone found in this study were lower than those found in studies that used blood (Toniolo *et al*, 1995; Berrino *et al*, 1996; Dorgan *et al*, 1996; Thomas *et al*, 1997; Hankinson *et al*, 1998; Cauley *et al*, 1999; Kabuto *et al*, 2000), but quite similar to the only other prospective study that used urine samples (Key *et al*, 1996). A recently pooled analysis of nine prospective studies on serum levels found a two-fold increased risk in the highest quintiles of oestrone, oestradiol and testosterone (The Endogenous Hormones and Breast Cancer Collaborative Group, 2002).

For elevated levels of the nonaromatisable androgen 3*α*D, we also found an increased risk for breast cancer. To our knowledge no prospective studies have examined the risk of breast cancer for circulating androgens that cannot be aromatised into oestrogens, such as dihydrotestosterone or its metabolites. However, one case–control study also showed an increased risk in postmenopausal women who had elevated levels of serum dihydrotestosterone (highest *vs* lowest quartile: OR=2.0, 95% CI: 0.8–5.0) and urinary androstanediol (highest *vs* lowest quartile: OR=3.4, 95% CI: 1.4–8.7) (Secreto *et al*, 1991). In addition, we found that women with high levels of androgens but low levels of oestrogens had an increased risk. These results suggest that, apart from conversion into oestrogens, androgens may also have a direct effect on breast cell proliferation and therefore on breast cancer risk, in line with results from *in vitro* studies (Maggiolini *et al*, 1999).

Since adipose women have a higher conversion from androgens into oestrogens in the adipose tissue, it has been hypothesised that BMI might actually be in the aetiological pathway, rather than act as a confounder. This may be the case for the reproductive risk factors as well (Bernstein and Ross, 1993). Adjustment for these risk factors would then lead to overcorrection of the results. In line with the pooled analysis, neither BMI nor any of the reproductive factors altered the risks found for any of the hormones. ‘Family history of breast cancer’ was the only confounder in our study; it was more prevalent among breast cancer cases, and women with a positive family history showed higher levels of (especially) oestrone and testosterone (statistically nonsignificant). Excluding women with such a positive family history from the analyses did not change the results (data not shown).

In conclusion, this study, the largest prospective study on endogenous hormones and breast cancer risk so far, shows that women with higher levels of oestrogens and androgens after menopause have an increased risk for breast cancer compared to women with lower concentrations.
